# Phase I study of cord blood transplantation with intrabone marrow injection of mesenchymal stem cells: A clinical study protocol

**DOI:** 10.1097/MD.0000000000010449

**Published:** 2018-04-27

**Authors:** Tatsunori Goto, Makoto Murata, Seitaro Terakura, Tetsuya Nishida, Yoshiya Adachi, Yoko Ushijima, Kazuyuki Shimada, Yuichi Ishikawa, Fumihiko Hayakawa, Nobuhiro Nishio, Satoshi Nishiwaki, Akihiro Hirakawa, Katsuyoshi Kato, Yoshiyuki Takahashi, Hitoshi Kiyoi

**Affiliations:** a Department of Hematology and Oncology, Nagoya University Graduate School of Medicine; b Center for Advanced Medicine and Clinical Research, Nagoya University Hospital; c Department of Pediatrics, Nagoya University Graduate School of Medicine, Nagoya, Aichi, Japan.

**Keywords:** cord blood transplantation, engraftment, graft-versus-host disease, intrabone marrow injection, mesenchymal stem cell

## Abstract

**Introduction:**

Delayed hematological recovery, graft failure, and acute graft-versus-host disease (GVHD) still remain major problems in cord blood transplantation (CBT). Mesenchymal stem cells (MSCs) are known to support bone marrow stroma and promote hematopoiesis. Additionally, MSCs possess immunomodulatory properties and are used clinically for the treatment of acute GVHD. Therefore, the use of MSCs to enhance engraftment and prevent GVHD after allogeneic hematopoietic cell transplantation has been explored. Recent clinical trials have shown the feasibility and safety of intravenous cotransplantation of MSCs with cord blood cells in pediatric patients, but not in adult patients, who are at greater risk of graft failure. As for the route of administration of MSCs, direct intrabone marrow injection of MSCs is thought to enhance the engraftment of cord blood cells more than intravenous injection. Based on these background findings, this clinical trial was designed to develop a new strategy to enhance engraftment and prevent GVHD after CBT.

**Methods and analysis:**

This is a single-center, phase I, clinical study to evaluate the safety of CBT combined with intrabone marrow injection of ex vivo expanded MSCs from bone marrow of a third-party donor. Adult patients with hematological disorders are eligible for this study. The target sample size is 5, and the registration period is 3 years. The target dose of MSCs infused is 0.5 × 10^6^ cells/kg of patient body weight. On the day of CBT, MSCs are injected into the intrabone marrow of the patient 4 hours before the infusion of a single cord blood unit. The conditioning regimen varies according to patient age and disease. GVHD prophylaxis consists of a combination of tacrolimus and methotrexate. The primary endpoint of this study is infusional toxicity of MSCs within 14 days after transplantation.

## 1 Introduction

Cord blood transplantation (CBT) has been increasingly used as a curative treatment for various hematological disorders. However, delayed hematological recovery and a higher rate of graft failure after CBT lead to an increased risk of transplant-related mortality in the early period after transplant.^[1,2]^ To overcome these obstacles, several strategies, such as double-unit CBT,^[3]^ ex vivo expansion of cord blood-derived CD34^+^ cells,^[4–8]^ and intrabone marrow transplantation of cord blood cells,^[9,10]^ have been explored. Besides these approaches, cotransplantation of cord blood and mesenchymal stem cells (MSCs) has recently been reported.^[11–14]^

MSCs are a heterogeneous subset of stromal stem cells and can be isolated from many tissues, such as bone marrow, adipose tissue, cord blood, and placenta. MSCs have the capacity for self-renewal and can differentiate into mesodermal lineage cells.^[15]^ In bone marrow, MSCs differentiate into bone-marrow stroma cells, osteocytes, osteoblasts, and endothelial cells. All of these cells form the bone marrow microenvironment, known as the hematopoietic stem cell niche, and support hematopoiesis.^[16,17]^ Besides this hematopoietic support capacity, MSCs can modulate immune responses by producing several cytokines and growth factors,^[15]^ and this immunomodulatory property of MSCs has already been applied to the treatment of graft-versus-host disease (GVHD) after allogeneic hematopoietic cell transplantation (HCT).^[18,19]^ In addition, MSCs are able to evade allogeneic rejection because of low expression levels of HLA molecules and no expression of costimulatory molecules, such as CD80, CD86, and CD40.^[20,21]^ Furthermore, MSCs can be easily expanded ex vivo and stored by cryopreservation. Therefore, ex vivo expanded and cryopreserved MSCs derived from a third-party donor can be used for patients without considering HLA matching. Because of these properties, MSCs have been explored for application in enhancing engraftment and preventing GVHD after allogeneic HCT.

The feasibility and safety of intravenous cotransplantation of MSCs with cord blood cells in pediatric patients have been reported by 4 clinical trials.^[11–14]^ However, cotransplantation of MSCs and cord blood cells has not been evaluated in adult patients, who are at greater risk of graft failure because of a lower cord blood cell dose per patient body weight. As for the route of MSC administration, several animal model experiments have shown that MSCs infused intravenously were trapped in lung,^[22,23]^ and direct intrabone marrow injection of MSCs could enhance the engraftment of transplanted cord blood cells more than intravenous injection.^[24]^ Additionally, intrabone marrow injection of MSCs has been reported to be safe in previous clinical studies.^[25,26]^

Based on these background findings, to develop a new strategy not only to enhance engraftment but also to prevent GVHD after CBT, a first phase I clinical trial was designed to evaluate the safety of CBT combined with intrabone marrow injection of ex vivo expanded MSCs for adult patients with hematological disorders.

## 2 Methods and analysis

### 2.1 Study design and setting

This study is a single arm, nonrandomized, open-label, single-center, phase I trial to evaluate the safety of CBT combined with intrabone marrow injection of MSCs generated from bone marrow of a third-party donor. The target sample size is 5, and the registration period is 3 years. The compliance of this study with the Act on the Safety of Regenerative Medicine was confirmed by the Ministry of Health, Labour and Welfare of Japan (number PA8160004, the latest edition ver. 5.1 22/Nov/2016). This study was registered with the University Hospital Medical Information Network Clinical Trials Registry (UMIN-CTR, number 000024291). Written informed consent will be obtained from all patients and donors before registration, in accordance with the Declaration of Helsinki. Patients and donors will be registered in this study after independent review by the data center in the Department of Hematology and Oncology, Nagoya University Graduate School of Medicine. Independent monitoring will be planned according to the Japanese clinical trial guideline at least annually.

### 2.2 Patient eligibility

The inclusion and exclusion criteria of the patients are listed in Table 1.

**Table 1:**
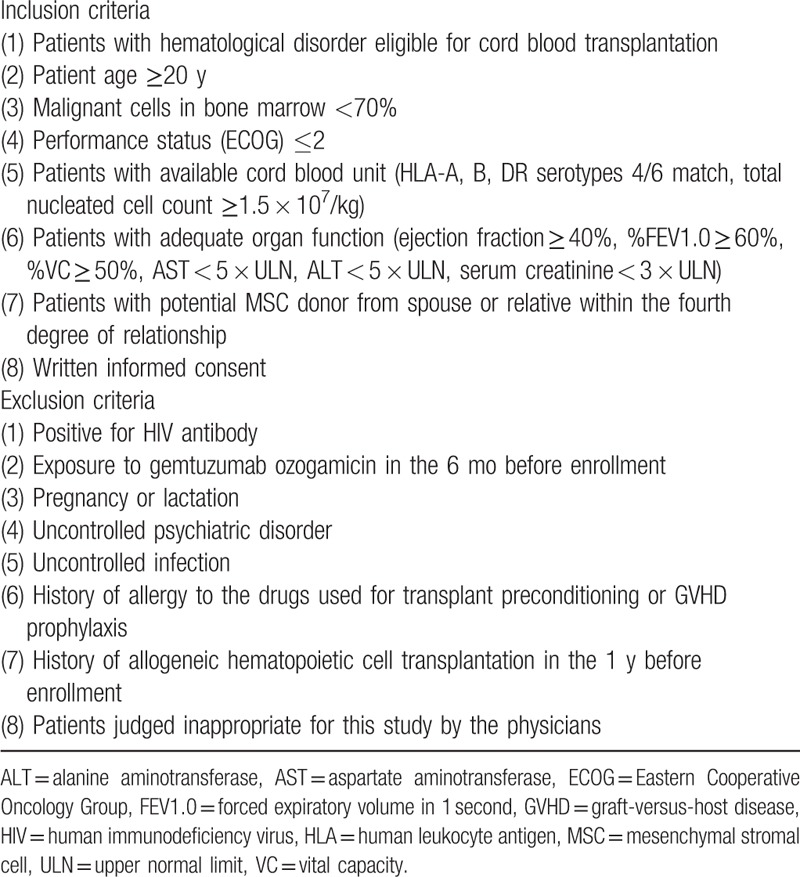
Patient eligibility.

### 2.3 Preparation of MSCs

The inclusion and exclusion criteria of the MSC donors are listed in Table 2. Bone marrow is harvested from the posterior iliac crest of the MSC donor with local anesthesia by the standard procedure of bone marrow aspiration. The target MSC dose is 0.5 × 10^6^ cells/kg of patient weight. The volume of bone marrow aspirate is determined according to patient weight; if the patient weight is <35, 35 to 50, or ≥50 kg, the volume of bone marrow aspirate is 10, 15, or 20 mL, respectively. Mononuclear cells are isolated by centrifugation of bone marrow using Ficoll-Paque PREMIUM (GE Healthcare Japan, Tokyo, Japan). The separated mononuclear cells are seeded in T-25 cell culture flasks at 1.0 to 2.0 × 10^7^ cells/flask in D-MEM medium (Thermo Fisher Scientific, Waltham, MA) containing 5% human platelet lysate and 2 IU/mL heparin (Mochida Pharmaceuticals, Tokyo, Japan) (referred to as culture medium) and cultured at 37°C in a humidified incubator containing 5% CO_2_. After culturing for 3 or 4 days, nonadherent cells are removed, and the adherent cells are further cultured. Cells are harvested at subconfluent using TrypLE Select (Invitrogen, Carlsbad, CA). Cells at passage 1 are seeded in the same number of T-75 culture flasks from T-25. Cells at passage 2 are seeded in the same number of T-225 culture flasks from T-75. Cells at passage 3 or 4 are seeded in the threefold number of T-225 flasks. MSCs are harvested after passage 3 or 4 according to the cell count, sampled, and cryopreserved at −150°C in CP-1 (Kyokuto Pharmaceutical, Tokyo, Japan) until the day of CBT.

**Table 2:**
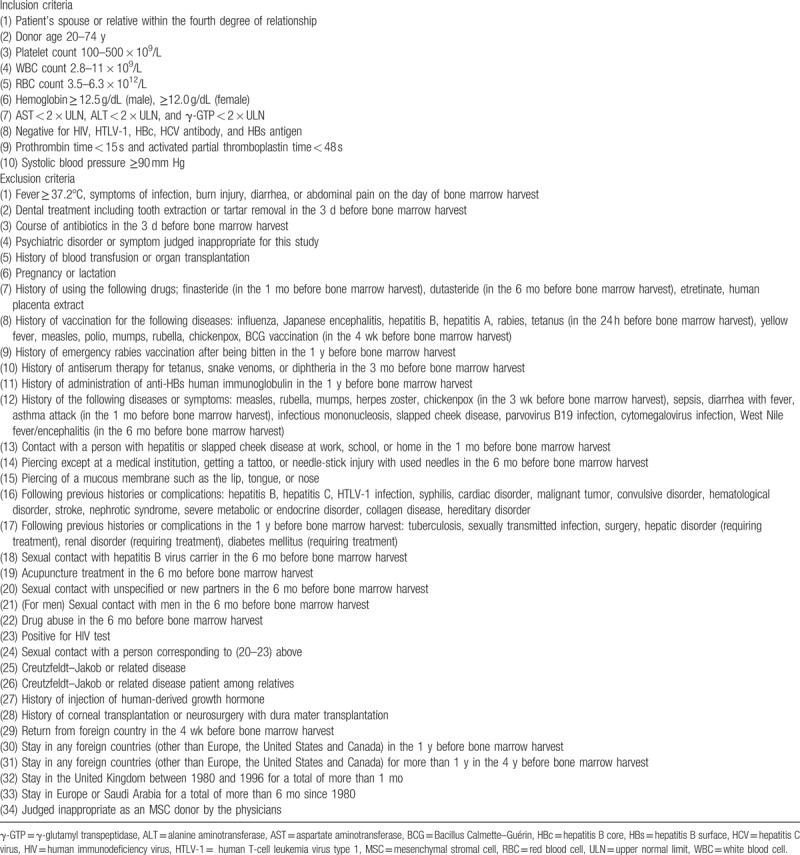
MSC donor eligibility.

Human platelet lysates are prepared from single-donor platelet concentrate provided by the Japan Red Cross Blood Center by the Application for the use of blood donated in Japan based on the “Guidelines on the use of donated blood in R&D, etc.”. Platelet concentrate is frozen at −30°C and thawed twice and then stored at −30°C. The frozen platelet concentrate is thawed at 4°C and centrifuged to obtain supernatant as platelet lysate.

Criteria for release of MSCs for clinical use are as follows: viability ≥70%, viable cell count ≥0.2 × 10^6^ cells/kg of patient weight, absence of contamination by pathogens (as documented by sterility test, endotoxin test, β-d-glucan assay, mycoplasma PCR test, and viral PCR tests for hepatitis B and C virus, human immunodeficiency virus type 1, parvovirus B19, herpes simplex virus, varicella-zoster virus, human herpesvirus-6, cytomegalovirus, Epstein–Barr virus), and immune phenotype characterized by the expression of CD73, CD90, and CD105 surface molecules (≥90%) and the absence of CD14, CD19, CD34, CD45, and HLA-DR expressions (≤10%).

### 2.4 Transplant procedure

The conditioning regimen is not defined in this study. In vivo purging of T cells using treatments such as antithymocyte globulin is prohibited. GVHD prophylaxis consists of the combination of tacrolimus and short-term methotrexate. Granulocyte colony-stimulating factor is administered from 7 days after transplantation until neutrophil engraftment.

### 2.5 Cotransplantation of MSCs and cord blood cells

On the day of CBT, MSCs are thawed, washed, and resuspended in 2 to 10 mL of a saline solution. The premedication with hydrocortisone 100 mg and chlorpheniramine 10 mg is administered approximately 30 minutes before injection of MSCs. After local anesthesia, a standard bone marrow aspiration needle is inserted into the iliac bone on one side. To assess that the needle is securely inserted into the bone marrow cavity, aspiration of <0.5 mL bone marrow is done. Then, approximately 5 mL of MSC suspension are injected slowly. This procedure is repeated on the iliac bone on the contralateral side. Four hours after MSC injection, cord blood is infused intravenously with the standard procedure.

### 2.6 Endpoints

The primary endpoint of this study is infusional toxicity of MSCs within 14 days after transplantation. Infusional toxicity is defined as adverse events that could not be explained by other complications, such as regimen-related toxicity or infection, that generally occur after transplantation. Secondary endpoints include the rate of engraftment, the time to hematopoietic recovery, the incidences and severities of acute and chronic GVHD, the incidences of regimen-related toxicities and infection, and the probabilities of nonrelapse mortality (NRM) at 100 days, relapse, disease-free survival (DFS), and overall survival (OS) at 1 year after transplantation.

### 2.7 Definitions and statistical analysis

Engraftment is defined as neutrophil recovery greater than 0.5 × 10^9^/L for 3 consecutive days. The time to neutrophil engraftment is defined as the first day of achieving an absolute neutrophil count greater than 0.5 × 10^9^/L for 3 consecutive days. The time to platelet and reticulocyte recoveries is defined as the first days of achieving a platelet count more than 20 × 10^9^/L, and a reticulocyte count more than 1% for 3 consecutive days without transfusions. Primary graft failure is defined as the lack of neutrophil engraftment in patients surviving at least 60 days, and secondary graft failure is defined as neutrophil engraftment followed by a decline in neutrophil count to below 0.5 × 10^9^/L for 3 consecutive days. Acute GVHD is diagnosed and graded according to the consensus criteria.^[27]^ Chronic GVHD is evaluated according to the traditional Seattle criteria^[28]^ and the NIH criteria for diagnosis and severity of chronic GVHD.^[29]^ Relapse is defined as recurrence of disease after transplantation. NRM is defined as death without disease relapse. The probabilities of relapse, NRM, acute GVHD, and chronic GVHD are estimated on the basis of cumulative incidence curves to accommodate the following competing events: death without relapse for relapse, relapse for NRM, and death without GVHD and relapse for GVHD. The probability of DFS is calculated from the day of transplantation until death from any cause, relapse, or last follow-up according to the Kaplan–Meier method. The probability of OS is calculated from the day of transplantation until death from any cause or last follow-up according to the Kaplan–Meier method.

## 3 Discussion

This clinical phase I trial has been designed to evaluate the safety of CBT combined with intrabone marrow injection of ex vivo expanded MSCs. We consider that coinfusion of MSCs, which are known to promote hematopoiesis and modulate immune reaction, will both enhance the engraftment of cord blood cells and prevent the occurrence of GVHD. This strategy might be applicable not only to CBT, but also bone marrow or peripheral-blood stem-cell transplantation, leading to benefits such as suppression of severe GVHD in HLA-mismatched settings and reduction of the burden imposed on hematopoietic stem cell donors by decreasing the required stem cell number. We hope that this study will provide a basis for further clinical trials of this strategy for the improvement of the outcome of hematopoietic cell transplantation.

## Author contributions

**Conceptualization:** Tatsunori Goto, Makoto Murata.

**Funding acquisition:** Makoto Murata.

**Methodology:** Tatsunori Goto, Makoto Murata, Seitaro Terakura, Tetsuya Nishida, Yoshiya Adachi, Yoko Ushijima, Kazuyuki Shimada, Yuichi Ishikawa, Fumihiko Hayakawa, Nobuhiko Nishio, Satoshi Nishiwaki, Akihiro Hirakawa, Katsuyoshi Kato, Yoshiyuki Takahashi, Hitoshi Kiyoi.

**Writing — original draft:** Tatsunori Goto, Makoto Murata.

**Writing — review and editing:** Tatsunori Goto, Makoto Murata, Seitaro Terakura, Tetsuya Nishida, Yoshiya Adachi, Yoko Ushijima, Kazuyuki Shimada, Yuichi Ishikawa, Fumihiko Hayakawa, Nobuhiko Nishio, Satoshi Nishiwaki, Akihiro Hirakawa, Katsuyoshi Kato, Yoshiyuki Takahashi, Hitoshi Kiyoi.
